# Vincoside Lactam from Edible *Uncaria rhynchophylla*: A Food-Derived Indole Alkaloid for Dietary Postprandial Glycemic Regulation and Functional Food Development

**DOI:** 10.3390/foods15183329

**Published:** 2026-09-20

**Authors:** Haoshu Liu, Hairong Xiang, Huannan Li, Ruyu Jiang, Yue Zhang, Linfeng Zhao, Dawei Zeng, Jiazhen Xie, Yanju Gong, Xiongbin Chen, Lan Yang

**Affiliations:** 1 School of Clinical Medicine, Chengdu University of Traditional Chinese Medicine, No. 1166, Liutai Avenue, Wenjiang District, Chengdu 611137, China; liuhaoshu6571@163.com; 2 School of Basic Medical Sciences, Chengdu University of Traditional Chinese Medicine, No. 1166, Liutai Avenue, Wenjiang District, Chengdu 611137, China; 13036697356@163.com (H.X.); ruyujiang@163.com (R.J.); zhangyue2001yy@outlook.com (Y.Z.); zlinfengzhao@hotmail.com (L.Z.); zengdawei911@outlook.com (D.Z.); jiazhen18796@outlook.com (J.X.); gongyanju@cdutcm.edu.cn (Y.G.); 3 Hubei Engineering Institute, Huangshi 435000, China; lihn0722@163.com

**Keywords:** α-glucosidase inhibitor, vincoside lactam (VCS-LT), molecular docking, bioactive alkaloid, functional food, postprandial glycemic control

## Abstract

α-Glucosidase inhibitors play a role in postprandial blood glucose. However, synthetic hypoglycemic drugs often cause gastrointestinal discomfort and are unsuitable for use as food additives. Natural compounds from plants have therefore attracted interest as safer candidates for hypoglycemic functional foods. Vincoside lactam (VCS-LT), an indole alkaloid isolated from the leaves of *Uncaria rhynchophylla* (Miq.) Miq. ex Havil., is traditionally used in East Asian herbal teas and dietary supplements. We evaluated its carbohydrate-digestion inhibitory activity to provide experimental evidence for the development of herbal beverages and grain supplements with hypoglycemic properties. The in vitro assay used yeast-derived α-glucosidase, whereas the molecular docking targeted human maltase-glucoamylase (MGAM)—a distinction that is important for interpreting the respective findings. Molecular docking indicated the stable binding of VCS-LT to two catalytic domains of maltase-glucoamylase, a key intestinal enzyme involved in reducing postprandial glucose excursion after maltose loading following the consumption of high-carbohydrate staple foods. In a p-nitrophenyl-α-D-glucopyranoside-based assay using yeast-derived α-glucosidase, VCS-LT showed potent inhibitory activity. In alloxan-induced diabetic mice, oral VCS-LT reduced fasting glucose and attenuated postprandial spikes after maltose loading. In contrast to the gastrointestinal side effects commonly associated with synthetic α-glucosidase inhibitors such as acarbose, no gastrointestinal discomfort or other adverse effects were observed during the 12-day administration period. Collectively, our findings demonstrate that VCS-LT is a potent food-derived α-glucosidase inhibitor that effectively attenuates postprandial glucose excursion in diabetic mice, which supports its potential application as a functional food ingredient for daily dietary glycemic management. Nevertheless, our work remains at the proof-of-concept level, and future studies on processing stability, bioaccessibility, food-matrix interactions, sensory acceptability, and safety at food-use levels are essential before any commercial application can be envisioned.

## 1. Introduction

Diabetes mellitus is a metabolic disease that is spreading rapidly worldwide. The 11th edition of the IDF Diabetes Atlas estimates that in 2024, approximately 589 million adults aged 20–79 years were living with diabetes, and the condition caused around 3.4 million deaths globally [1]. The severe burden of this disease has pushed the search for effective pharmacological treatments for diabetes.

α-Glucosidase inhibitors (AGIs) target the specific enzymes sucrase and maltase at the brush border of the small intestine. This inhibitory effect delays the breakdown of carbohydrates and helps control postprandial blood glucose levels [2,3]. AGIs encompass a broad class of compounds that target various intestinal glucosidases, including maltase-glucoamylase (MGAM) and sucrase-isomaltase. Among these, MGAM is of particular interest because it catalyses the final step of starch digestion and converts maltose and maltotriose into absorbable glucose. In this study, we therefore selected MGAM as the representative target for molecular docking to predict the binding mode of vincoside lactam (VCS-LT) to this specific intestinal enzyme, with a 4-nitrophenyl-α-D-glucopyranoside (pNPG)-based assay used to evaluate its general α-glucosidase inhibitory activity.

The rapid increase in diabetes prevalence has heightened demand for natural functional foods that target postprandial glycemia. Synthetic AGIs are limited in daily dietary interventions due to flatulence and diarrhoea following long-term intake. Bioactive compounds derived from edible medicinal plants now serve as clean-label additives for low-glycemic baked goods, herbal infusions, and nutritional powders to enable sustained mild glycemic regulation through routine dietary intake [4,5,6].

Historical records, including the Bencao Gangmu Shiyi, reveal that tender leaves were steamed and dried for daily tea consumption in Fujian and Zhejiang provinces to ‘clear the heart and brighten the eyes’ [7]. *U. rhynchophylla* leaves are traditionally used as a herbal tea ingredient in China and Southeast Asia and incorporated into compound plant drinks and solid dietary supplements. VCS-LT, its dominant indole alkaloid, is thought to act as a dietary hypoglycemic bioactive substance by blocking intestinal starch digestion. Reports have confirmed that *Uncaria* alkaloids possess an α-glucosidase inhibitory capacity [8], yet monomeric VCS-LT has only been purified in a few studies to evaluate its potential as a standardised functional food additive [9].

VCS-LT isolated from the leaves of *U. rhynchophylla* is one of the main indole alkaloid components [10] and has the potential to improve glucose homeostasis. Studies have shown that monoterpenoid indole alkaloids such as vincamine can protect pancreatic β-cells and lower blood glucose levels in diabetic mice, thus providing a structural basis for the antidiabetic activity of VCS-LT [11]. Various *Uncaria* species possess antidiabetic and antioxidant properties, and their alkaloid constituents are considered the major active components responsible for regulating glucose metabolism [12,13].

The current approaches used to evaluate α-glucosidase inhibitory activity primarily include in vitro and in vivo assays. Common in vitro methods involve the use of pNPG or sugars as substrates, immobilised enzyme screening, high-throughput screening, and computational ligand-based virtual screening [8,14].

Although in vitro assays offer advantages, such as higher speed, lower cost, and clearer mechanistic insights, over in vivo assays, they are susceptible to false positives and cannot fully replicate in vivo conditions. Accordingly, we first evaluated VCS-LT in vitro, after performing the in vitro assays, we moved to in vivo validation to exclude false positives and determine the true hypoglycemic potential of VCS-LT. Alloxan (ALX) and streptozotocin are widely used chemical agents for inducing hyperglycemic mouse models [15,16]. Both selectively damage pancreatic β-cells, although streptozotocin is less frequently employed due to its higher cost, poorer model stability, and shorter duration of action [17].

We systematically evaluated the α-glucosidase inhibitory and hypoglycemic effects of VCS-LT, a natural product isolated from *U. rhynchophylla*, using an optimised pNPG-based assay, molecular docking, and an ALX-induced diabetic mouse model to provide experimental support for developing novel hypoglycemic functional food formulations based on *Uncaria* extracts.

## 2. Materials and Methods

### 2.1. Materials and Instruments

#### 2.1.1. Animals

Equal numbers of male and female SPF-grade Kunming mice (15–17 g) (3 males and 3 females per group) were supplied by Beijing Sibeifu Biotechnology Co., Ltd. (Beijing, China) (ethics approval number: 2024157). The mice were housed at our university, acclimatised for 3 d, and had free access to standard chow and purified water.

#### 2.1.2. Drugs and Reagents

VCS-LT (CAS number 23141-27-7, HPLC purity ≥ 98%, Cat. number JOT-10861) was purchased from Chengdu Victory Biotechnology Co., Ltd. (Chengdu, China). During the cell treatment, the final dimethyl sulfoxide (DMSO) concentration was maintained below 0.1% (*v*/*v*) to eliminate potential solvent cytotoxicity. Acarbose (Glucobay, HY-B0089) was purchased from Bayer HealthCare AG (Leverkusen, Germany). Maltose and α-glucosidase (from Saccharomyces cerevisiae) were obtained from Beijing Solarbio Science & Technology Co., Ltd. (Beijing, China) pNPG (N1377) and ALX (A7413) were bought from Sigma-Aldrich Corporation (St. Louis, MO, USA).

#### 2.1.3. Instruments

The following instruments were used: a biochemical incubator (Model LRH-250, Shanghai Yiheng Scientific Instruments Co., Ltd., Shanghai, China), an electronic analytical balance (Model BSA124S-CW, Sartorius Scientific Instruments (Beijing) Co., Ltd., Beijing, China), a blood glucose meter (Model OGM-111, Acon Biotech Co., Ltd., Hangzhou, China), and a microplate shaker (No. 270400, Boekel Scientific, Feasterville-Trevose, PA, USA).

### 2.2. Solution Preparation

1. VCS-LT stock solution: We dissolved 1 g of purified VCS-LT in 6.4 mL DMSO, which was stored at −20 °C.
2. VCS-LT solution for the in vitro experiments: The thawed stock solution was then diluted with phosphate-buffered saline (PBS) (pH 7.0) to 100 μg/mL (stored at −20 °C).
3. VCS-LT solution for the in vivo experiments: We combined 2.5 mg Tween-80 with 320 μL of the thawed stock solution, and 5 mL of this mixture was diluted with purified water.
4. Acarbose purification: Fifteen Glucobay tablets were ground to a fine powder and dissolved in 10 mL of distilled water. After centrifugation at 3000× *g* for 10 min at 4 °C, the supernatant was retained. The extraction was repeated once with 6 mL of distilled water.
5. Acarbose solution for the in vitro experiments: We dissolved 0.0010 g of purified acarbose in 1 mL PBS (pH 7.0), −20 °C.
6. Acarbose solution for the in vivo experiments: We mixed 0.0500 g of purified acarbose with 320 μL DMSO and 32 mg of Tween-80 and diluted this to 5 mL with pure water.
7. α-Glucosidase solution: We prepared 0.0025 g in 1.5 mL of freshly made PBS (pH 7.0) containing 0.2% azide buffer solution (ABV) immediately prior to use.
8. ABV: We dissolved 0.0040 g of ABV in 2 mL of PBS (pH 7.0), which was freshly prepared prior to use.
9. pNPG solution: We prepared 0.0301 g of pNPG in 10.0 mL of PBS (pH 7.0), and this was stored at −20 °C for later use.
10. Sodium carbonate solution: We used 10 mL of PBS (pH 7.0) to dissolve 1.0599 g of anhydrous sodium carbonate, and the solution was kept at −20 °C for later use.
11. ALX solution: We dissolved 3.0000 g of ALX in 15 mL of normal saline. This was freshly prepared, protected from light, and stored at 2–6 °C.
12. Maltose solution: We dissolved 3.0000 g of maltose in 15.0 mL of distilled water.

### 2.3. Inhibitory Activity of VCS-LT Against α-Glucosidase In Vitro

#### 2.3.1. Determination of α-Glucosidase Inhibitory Activity

The α-glucosidase used in this assay was derived from Saccharomyces cerevisiae. This enzyme is commonly used as a surrogate in initial inhibitor screening due to its availability and active-site conservation with mammalian α-glucosidases. However, it is not identical to human intestinal MGAM.

Preliminary experiments confirmed that VCS-LT showed UV absorption that could interfere with absorbance measurements at 405 nm. Four experimental groups were therefore established to eliminate such interference: VCS-LT group, acarbose group, control group, and blank group. To reduce any edge effects caused by environmental fluctuations, 140 μL PBS (pH 7.0) was added to all 36 outer wells of the 96-well microplate as a buffer.

VCS-LT group: We added 20 μL PBS (pH 7. 0), 20 μL α-glucosidase solution, and 20 μL diluted VCS-LT solution (100 μg/mL) to each well and mixed them thoroughly on a microplate shaker. We then added 10 μL pNPG solution, which was mixed briefly and maintained at 37 °C for 40 min. The reaction was stopped by adding 80 μL of Na_2_CO_3_ solution, and the absorbance was read at 405 nm.

Acarbose group (100 μg/mL): We added 20 μL acarbose instead of VCS-LT. The remaining procedures were consistent with those for the VCS-LT group.

Control group: We used 20 μL PBS to replace the α-glucosidase. The other operations remained unchanged.

Blank group: The VCS-LT was substituted with 20 μL PBS. All the other steps were identical.

Based on the optical density (OD) value detection at 405 nm, the α-glucosidase inhibition rate of the VCS-LT group was calculated using Equation (1). All the experiments were performed in triplicate and averaged. The relative inhibition rate of the VCS-LT compared with the positive control acarbose was calculated using Equation (2) to evaluate its inhibitory potency.

(1) $$\mathrm{Inhibition}\;\mathrm{Rate}\;(\%)=[1-\frac{(OD_{\mathrm{Vincoside}\;\mathrm{lactam}}-OD_{\mathrm{Control}})}{(OD_{\mathrm{Blank}}-OD_{\mathrm{Control}})}]\;\times \;100\%\;$$

(2) $$\mathrm{Relative}\;\mathrm{Inhibition}\;\mathrm{Rate}\;(\%)=\frac{{\mathrm{Inhibition}\;\mathrm{Rate}}_{\mathrm{Vincoside}\;\mathrm{lactam}}}{{\mathrm{Inhibition}\;\mathrm{Rate}}_{\mathrm{Acarbose}}}\;\times \;100\%\;$$

#### 2.3.2. Virtual Molecular Docking Analysis

Molecular docking predicts the binding mode and free energy between a receptor macromolecule and a small-molecule ligand, which helps clarify the function and mechanism of the ligand. In the case of VCS-LT, molecular docking predicts the binding mode, sites, and free energy between VCS-LT and the target receptor, thus providing mechanistic insights into its inhibitory activity [18]. In this study, MGAM was selected as the target receptor because, although MGAM is less abundant than sucrase in the human intestinal brush border, it demonstrates significantly higher catalytic activity [19].

AutoDock Vina, version 1.2.0 (Center for Computational Structural Biology, Scripps Research Institute, La Jolla, CA; https://vina.scripps.edu/, accessed on 13 August 2026) was used to conduct the molecular docking between the VCS-LT conformations and the crystal structures of the MGAM N-terminal (MGAM-N, PDB ID: 3TOP) and C-terminal (MGAM-C, PDB ID: 2QMJ) domains [20]. The grid box was centred on the active site of each receptor, with the dimensions adjusted to fully cover the binding pocket. The optimal binding sites and docking parameters were identified, and the binding modes and binding energies between VCS-LT and the two terminals were predicted to interpret the molecular mechanism of VCS-LT against α-glucosidase. The crystal structure data used in the docking process were searched for in and retrieved from the Protein Data Bank (PDB).

### 2.4. Hypoglycemic Activity of VCS-LT In Vivo

#### 2.4.1. Establishment of an ALX-Induced Diabetic Mouse Model

ALX, which is widely applied to establish diabetes in animal models, selectively damages the pancreatic β-cells in animals by generating free radicals [21]. In this study, the mice weighing 20 ± 2 g were fasted for 8 h (water available) after acclimation for 3 d to obtain their baseline blood glucose measurements. After an 18 h fast (water available), the mice were weighed and injected intraperitoneally with ALX (120 mg/kg, freshly prepared) to trigger hyperglycemia. The control group received the same volume of normal saline. Within 24 h post-ALX injection, the drinking water for the model group was replaced with 20% glucose to prevent severe transient hypoglycemia and mortality. Four days later, following gavage and an 8-h fast, the baseline blood glucose levels of the mice were measured. The mice with a blood glucose ≥11.1 mmol/L were considered successfully induced hyperglycemic models.

#### 2.4.2. Grouping and Drug Administration

The diabetic mice were randomised into three groups, namely, the model, acarbose, and VCS-LT groups. A control group was also set. Drug administration started on day 0, following the ALX injection 5 days prior. The control and model groups received the same volume of the vehicle solution by oral gavage once daily. The acarbose and VCS-LT groups received 100 mg/kg of the respective compound by oral gavage once daily.

The fasting blood glucose (FBG) levels and oral maltose tolerance of the mice were monitored throughout the treatment period to determine the hypoglycemic efficacy of VCS-LT. At the end of the study, the mice were euthanised in accordance with the guidelines set by the Experimental Animal Ethics Committee.

#### 2.4.3. Observation Indicators and Detection Methods

##### General Physical Condition and Physiological Parameters

We assessed the activity, coat condition, and cage bedding dampness on a daily basis following diabetes induction and throughout the oral gavage period. Food and water intake and body weight were measured at days 0, 2, 6, and 11 relative to the initial gavage.

##### FBG

On days 0, 2, 6, and 11 following treatment, the mice were fasted for 8 h with water supplied ad libitum. Blood was sampled from the lateral tail vein, and FBG levels were determined using a portable glucometer.

##### Oral Maltose Tolerance Test (OMTT)

On day 12, after an overnight fast (8 h, water ad libitum), the baseline blood glucose (0 min) was measured. To perform an OMTT, an VCS-LT, acarbose, or vehicle solution was administered by oral gavage 30 min prior to maltose loading. All the mice then received the maltose solution (2.0 g/kg body weight) by oral gavage. The blood glucose levels were measured at 0, 0.5, 1, 2, and 3 h following maltose administration using a portable glucometer (GlucoCard II, ARKRAY, Kyoto, Japan) (Supplementary Figure S1 schematic experimental timeline).

##### Statistical Analysis

The values are presented as means ± standard deviation (SD) (*n* = 6). We used SPSS 24.0 (IBM Corp., Armonk, NY, USA) to analyse the data. Multi-group comparisons at each time point were performed using one-way ANOVA followed by Tukey’s post-hoc test. A *p*-value less than 0.05 was considered statistically significant.

## 3. Results

### 3.1. α-Glucosidase Inhibitory Activity In Vitro

#### 3.1.1. α-Glucosidase Inhibitory Activity

**Figure 1:**
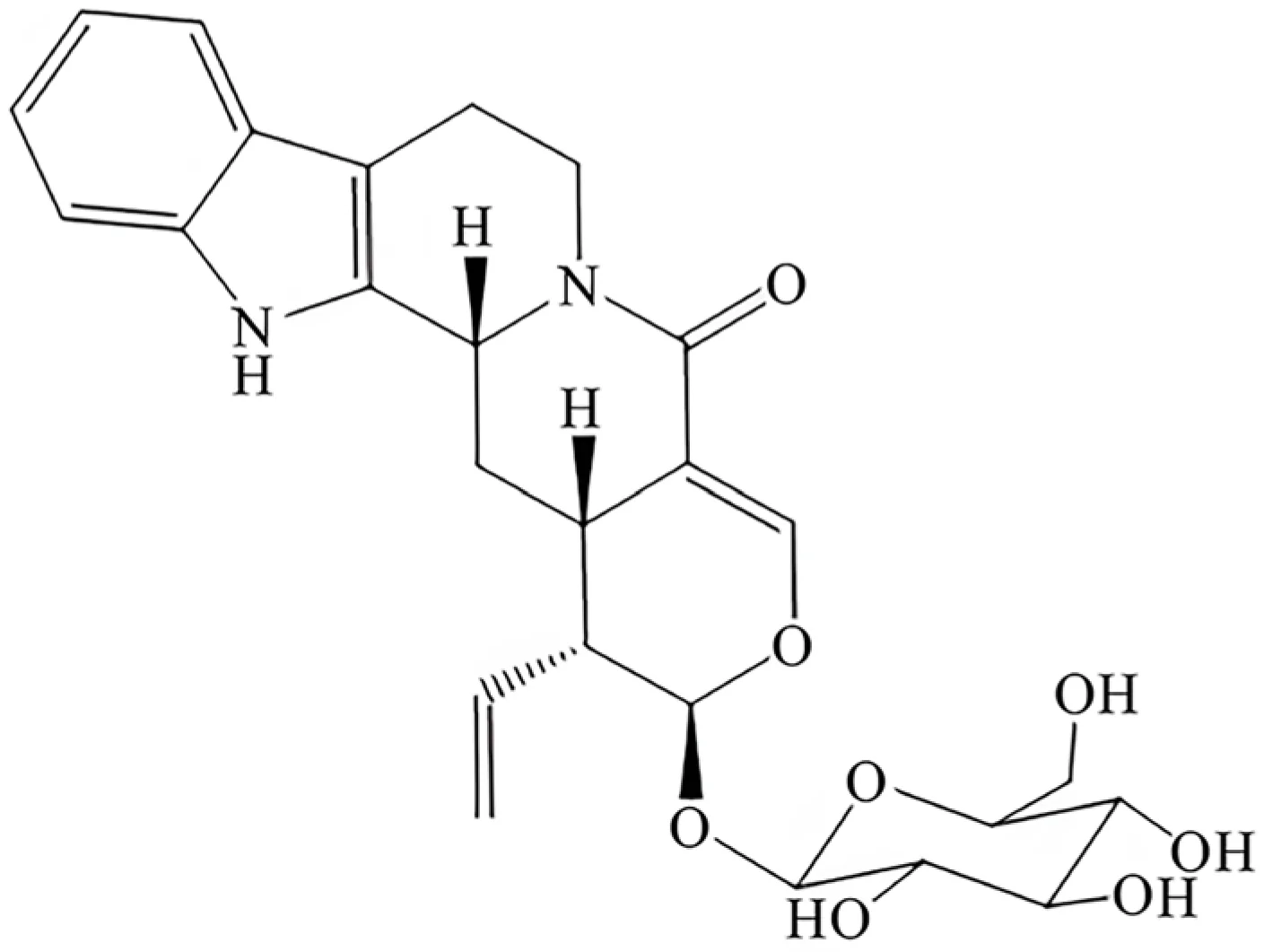
Chemical structure of vincoside lactam (VCS-LT).

We tested VCS-LT for α-glucosidase inhibition using the method described in Section 2.3.1. At 100 μg/mL, VCS-LT inhibited 47.47% of the enzyme activity, which corresponded to 95.06% of the inhibition observed with acarbose. Due to the limited concentration range tested (1–100 μg/mL), a reliable half-maximal inhibitory concentration (IC_50_) value could not be determined. Future studies with extended concentration ranges are thus needed to establish the true IC_50_ of VCS-LT (Figure 1).

Table 1 and Table 2 show how the VCS-LT and acarbose performed at different concentrations. Even at low doses (1, 10, and 50 μg/mL), VCS-LT maintained its activity, with inhibition rates above 35% (Table 2). Notably, VCS-LT retained more than 35% of its inhibitory activity at an ultra-low concentration (1 μg/mL).

#### 3.1.2. Virtual Molecular Docking

As shown in Table 3, the binding energies of VCS-LT with MGAM-C and MGAM-N were −9.5 kcal/mol and −9.1 kcal/mol, which were more negative than those of acarbose (−7.1 kcal/mol and −7.9 kcal/mol), respectively.

**Figure 2:**
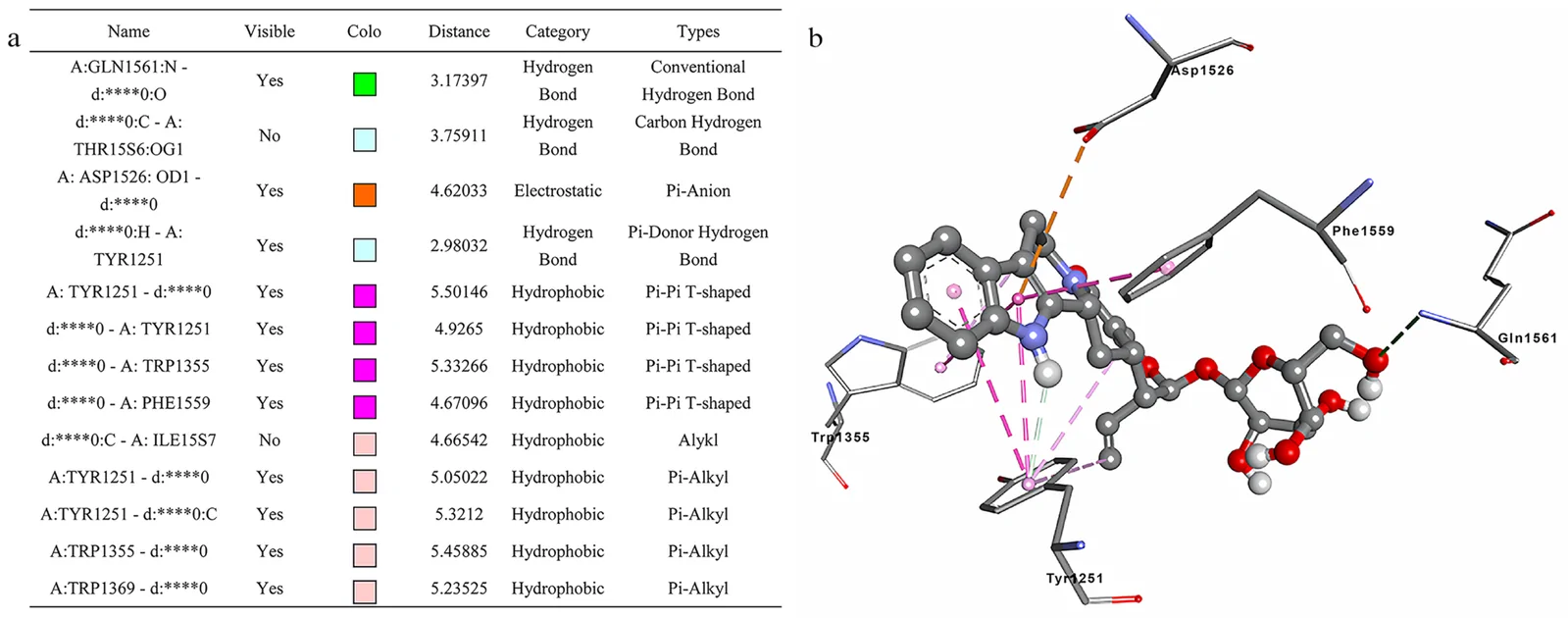
Binding mechanism of VCS-LT with the maltase-glucoamylase C-terminal (MGAM-C) domain. (**a**) Bonding information between VCS-LT and the MGAM-C active site. (**b**) Binding mode of VCS-LT at the active site of MGAM-C (PDB: 2QMJ). VCS-LT interacts with key pocket residues (for example, Trp1355, Tyr1251, and Gln1561).

To further explore the molecular binding mechanism, we present Figure 2, which illustrates the binding conformation and bonding information between VCS-LT and the MGAM-C active site. VCS-LT was anchored in the MGAM-C active pocket through hydrophobic interactions and hydrogen bonds.

VCS-LT established hydrogen bonds with three specific residues in MGAM-C: Tyr1251, Gln1561, and Trp1355. Among these, the bond with Tyr1251 was the shortest and exhibited the highest binding energy. This particular interaction played a crucial role in stabilising the complex. It also contributed significantly to the strong binding affinity of VCS-LT for MGAM-C.

**Figure 3:**
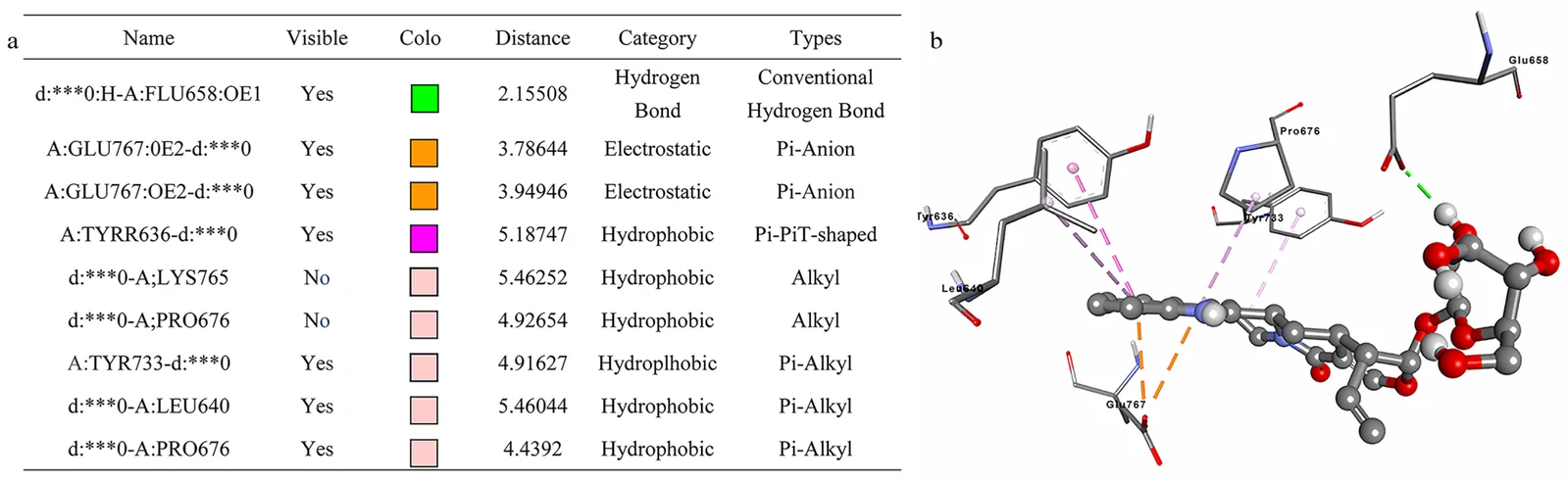
Binding mechanism of VCS-LT with MGAM-N. (**a**) Bond information for VCS-LT with the MGAM-N active site. (**b**) Binding mode of VCS-LT at the active site of MGAM-N (PDB: 3TOP). VCS-LT interacts with the key residues within the pocket, e.g., Tyr636, Tyr733, Glu658, thereby underlying its α-glucosidase inhibitory activity.

Figure 3 depicts the details of VCS-LT with the active site of MGAM-N. Among the residues, Glu658 had the shortest donor–acceptor distance and the highest binding energy.

### 3.2. Hypoglycemic Activity Study In Vivo

#### 3.2.1. Effects on General Physiological Phenotypes of Diabetic Mice

Intraperitoneal injection of ALX produced the characteristic diabetic phenotypes in the mice, which were clearly different from the control group. The diabetic mice showed weight loss, dull and ruffled fur, increased urine output with wet bedding, and reduced locomotor activity, which are hallmark features of the polyuria, polydipsia, polyphagia, and weight loss syndrome in diabetes mellitus. More importantly, consecutive oral gavage of VCS-LT (100 mg/kg) and acarbose (100 mg/kg) for 11 days led to the remarkable amelioration of all these phenotypes, with the treated mice showing full recovery of their fur lustre and fluffiness, increased spontaneous activity, and normal urine output.

#### 3.2.2. Body Weight Changes in Diabetic Mice

Table 4 provides a summary of the body weight changes in the mice while receiving treatment. The control group weighed significantly more than the model group at all time points (*p* < 0.05). The differences peaked on days 0, 6, and 11 (*p* < 0.01), which indicated successful diabetes induction and chronic hyperglycemia-related growth impairment. The baseline weights between the groups differed (model vs. VCS-LT: *p* < 0.05). To account for these baseline differences, subsequent weight recovery was evaluated by analysing the percentage change from the baseline rather than absolute values (*p* > 0.05).

Although the rates were different, all the groups gained weight following treatment. The control group showed the largest increase, which exceeded those of all the diabetic groups. The VCS-LT group gained more weight than the acarbose and model groups, with the body weight values of the VCS-LT group gradually approaching those of the control group. The body weight responses varied within the model group. Most of the mice lost weight, while a few showed spontaneous glycemic recovery with concomitant weight gain. This heterogeneity was not observed in the VCS-LT group.

#### 3.2.3. Hypoglycemic Efficacy of VCS-LT on FBG in Diabetic Mice

**Figure 4:**
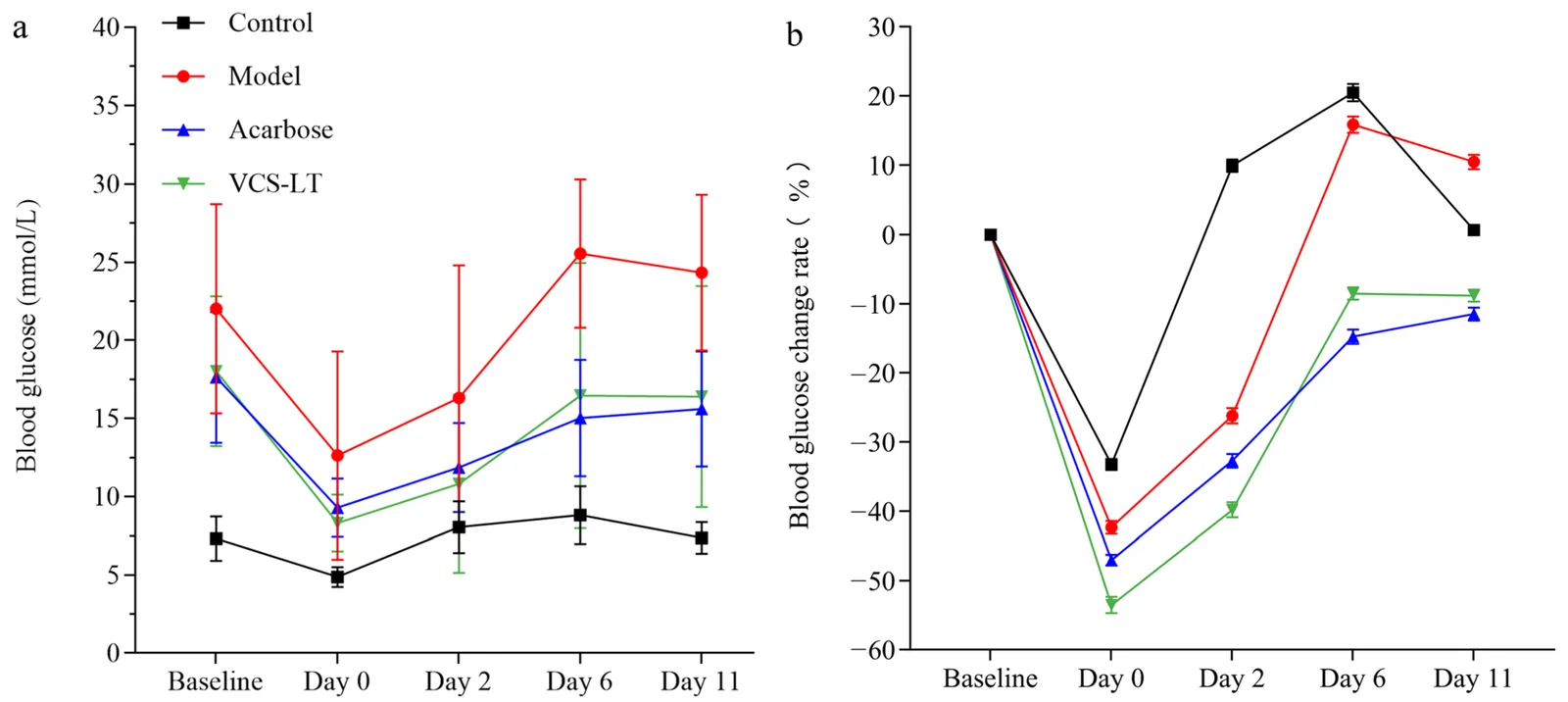
Effects of VCS-LT on fasting blood glucose (FBG) and its change rate in diabetic mice. (**a**) FBG levels at baseline and days 0, 2, 6, and 11 in the control, model, acarbose, and VCS-LT groups. (**b**) Percentage changes in FBG relative to the baseline values over the experimental period. Mean ± SD (*n* = 6).

Figure 4 shows the baseline FBG levels and changes in the mice while receiving treatment. Before the treatment, the FBG was higher in the model group than in the control group (*p* < 0.01), with no differences between the model, VCS-LT, and acarbose groups (*p* > 0.05), which confirmed successful diabetes induction.

The FBG levels decreased, then rebounded, and later stabilised across all the groups. Throughout the treatment, the FBG levels remained elevated in the model group relative to those of the control group (*p* < 0.01). The model group showed a transient FBG dip on days 0–2, followed by a rebound above baseline on days 6–11, a pattern that is consistent with β-cell dysfunction.

In both treatment groups, the FBG levels remained below baseline. On days 6 and 11, the FBG levels were lower in the VCS-LT and acarbose groups than in the model group (*p* < 0.05 and *p* < 0.01, respectively). The VCS-LT group also had slightly lower FBG levels than the acarbose group at all the measured time points, with no signs of hypoglycemic tolerance over the 12-day period.

#### 3.2.4. α-Glucosidase Inhibitory Activity of VCS-LT Assessed by OMTT

**Figure 5:**
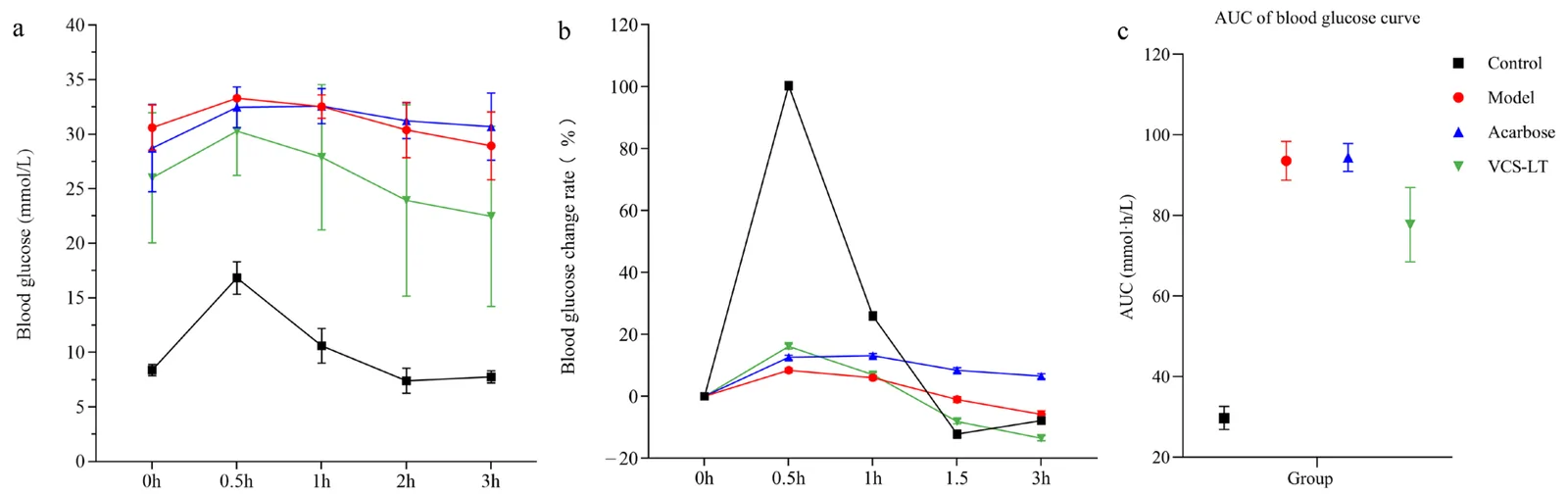
Effects of VCS-LT on the oral maltose tolerance test (OMTT) results among the diabetic mice. (**a**) Blood glucose levels measured at 0, 0.5, 1, 2, and 3 h after oral maltose administration. (**b**) Blood glucose change rate calculated relative to the baseline (0 h) values. (**c**) Area under the curve of the blood glucose–time curves. Mean ± SD (*n* = 6).

Figure 5 shows the blood glucose responses of the mice to maltose at 0.5, 1, 2, and 3 h. The blood glucose levels rose after the maltose gavage, peaked at 0.5 h, and then declined. The rate of decline followed: control > VCS-LT > model > acarbose. The blood glucose levels in the model group remained higher than those in the control group at all the time points (*p* < 0.01), which indicated sustained postprandial hyperglycemia. From 2 h to 3 h, the VCS-LT group had lower blood glucose levels than the model and acarbose groups (*p* < 0.05).

The area under the curve was 77.70 ± 23.64 in the VCS-LT group versus 93.54 ± 4.82 in the model group and 94.39 ± 4.33 in the acarbose group, which accounted for a 16.93% reduction in the VCS-LT group relative to the model group.

## 4. Discussion

### 4.1. In Vitro α-Glucosidase Inhibitory Activity and Molecular Docking

#### 4.1.1. Significant Effects and Comparison with Similar Compounds

α-Glucosidase catalyses the terminal hydrolysis of dietary carbohydrates into absorbable glucose and is therefore an important target for controlling postprandial hyperglycemia in people with diabetes mellitus [2,3]. In the present study, VCS-LT exerted concentration-dependent inhibitory activity against α-glucosidase, with greater inhibitory potency under the assay conditions.

Sixteen indole alkaloids were obtained from *U. rhynchophylla*. Among them, alkaloid 5 showed the strongest inhibition of α-glucosidase. It produced an IC_50_ of 18.45 ± 0.77 μM. Enzyme kinetics indicated that alkaloid 5 acts as a mixed-type inhibitor of α-glucosidase, and docking simulations supported this result. The docking score for alkaloid 5 with α-glucosidase reached −10.7 kcal/mol [22]. The indole alkaloids from *U. rhynchophylla* may therefore represent a source of AGIs. Nonetheless, VCS-LT showed a more favourable inhibition profile than alkaloid 5 at low concentrations. Alkaloid 5 inhibited α-glucosidase by only approximately 3% at the lowest tested concentration, while VCS-LT maintained more than 35% inhibition at concentrations of 1–50 μg/mL, indicating its stronger potential for glycemic regulation.

Synthetic acarbose is associated with intestinal side effects. In contrast, VCS-LT has a dietary origin and a tolerability profile that may benefit food-based applications. In our study, food-derived VCS-LT exhibited mild and balanced enzyme–inhibitory activity over 11 days of continuous oral administration, and no adverse gastrointestinal reactions were observed. This profile may support its long-term incorporation into daily dietary products intended to assist with glycemic management in populations with prediabetes.

VCS-LT maintained more than 35% inhibition at an ultra-low concentration. Relatively small amounts may therefore be required for incorporation into beverage- and cereal-based food matrices. The high purity and potent activity of VCS-LT suggest that only a small mass fraction of the compound (0.01% *w*/*w* at the tested dose) would be required in food matrices. This low incorporation ratio could help minimise production costs and reduce the risk of adverse effects on the sensory properties of food. Notably, the 100 mg/kg dose used in the current study was selected for direct comparison with acarbose at an equal mass dose. Future dose–response studies are thus warranted to determine whether lower doses can achieve comparable efficacy.

#### 4.1.2. Virtual Molecular Docking: Binding Mode and Binding Affinity of VCS-LT with MGAM

Molecular docking showed that VCS-LT interacted with the key residue Tyr1251 in the active pocket of MGAM-C. It also bound to Glu658 of MGAM-N. Previous research has documented the biological importance of these residues. Resin glycosides from Convolvulaceae have shown inhibitory activity inside the catalytic site near Tyr1251 [23]. The MGAM-N domain has also been identified as a critical binding site for AGIs [18]. α-Mangostin (AM) and its derivatives (AM 1–3), mangosteen (*Garcinia mangostana* L.) pericarp, and gnaphaffine A isolated from *Grewia optiva* J.R.Drumm. ex Burret forms hydrogen bonds and hydrophobic interactions with amino acid residues within α-glucosidase, and these interactions exert hypoglycemic activity against α-glucosidase [24,25].

The binding mode of VCS-LT differs from those of previously reported AGIs, as it interacts with the key residues Tyr1251 and Glu658 and forms an extensive hydrophobic network involving multiple residues, including Trp1355, Thr1586, and Gln1561. This broader interaction network may explain its lower binding energy, that is, −9.5 kcal/mol for MGAM-C and −9.1 kcal/mol for MGAM-N. In addition, it may account for its stronger inhibitory potency.

The predicted binding affinity of VCS-LT compared with those of synthetic agents supports its potential as a food-compatible AGI. The lower effective IC_50_ would minimise the dosage required in food matrices, thereby reducing production costs while maintaining efficacy. It should be noted that the molecular docking results in this study predicted binding to human MGAM, whereas yeast-derived α-glucosidase was used for the in vitro enzyme assay. These are complementary but distinct lines of evidence; the docking results should be interpreted as in silico predictions requiring experimental validation with the human enzyme.

### 4.2. Superior Efficacy and Mechanistic Validation of VCS-LT In Vivo

#### 4.2.1. Amelioration of Diabetic Symptoms

Body weight recovery is a key measure of antidiabetic treatment efficacy. In previous ALX-induced diabetic mouse models, a water-soluble polysaccharide from *Momordica charantia* L. was demonstrated to significantly ameliorate weight loss following continuous administration for 28 days at a high dose of 300 mg/kg [26].

In the present study, the diabetic mice in the acarbose and VCS-LT groups showed markedly reduced polydipsia and polyphagia during treatment. The mice in both treatment groups also exhibited gradual body weight recovery. More importantly, the VCS-LT group demonstrated a superior weight gain trend over the treatment period compared with the other groups. The other symptoms in the VCS-LT group similarly showed greater improvement than those in the acarbose group. It should be noted, however, that the baseline body weights were not fully comparable across all the groups. For instance, the VCS-LT group had a higher baseline weight than the model group. Accordingly, our interpretation of weight recovery was based primarily on the percentage change from baseline to minimise the impact of this baseline difference.

VCS-LT improved the health and survival of the mice in our study, likely by correcting the underlying metabolic disturbances. The SD data showed that the VCS-LT group had a larger SD than the acarbose group, which indicated greater inter-individual variability in weight recovery among the VCS-LT-treated mice compared with those in the acarbose group. The VCS-LT group showed relatively less variation than the model group, although the variation was greater than that in the acarbose group. This reflected the heterogeneity of the individual metabolic responses to VCS-LT. In contrast, the model group showed considerably greater variation than both the treatment groups. This pattern indicates that VCS-LT may have helped maintain metabolic stability and produced more consistent treatment outcomes than the untreated diabetic model.

Unlike synthetic acarbose, which is used as a drug, VCS-LT is derived from *U. rhynchophylla*, a plant with a history of use in East Asian teas. This food-compatible origin, together with its efficacy in promoting weight recovery and alleviating symptoms of diabetes, supports the potential of VCS-LT as a functional ingredient in low-glycemic food products. The dose required to demonstrate its efficacy appeared well tolerated during administration in this study, which suggests that VCS-LT could be incorporated into routine dietary patterns without the tolerability issues associated with synthetic alternatives. These findings establish VCS-LT as a food-derived AGI with potential for daily dietary support in individuals with prediabetes and diabetes. Its efficacy and tolerability profile support its use as a functional ingredient in everyday foods to help maintain postprandial glucose homeostasis.

#### 4.2.2. Significance of FBG Reduction

FBG is an important indicator for monitoring diabetes progression. In this study, the transient decrease during days 0–2 was likely associated with a nonspecific response to gavage. The subsequent increase above the baseline during days 6–11 was consistent with progressive β-cell dysfunction and confirmed the stable establishment of the ALX-induced diabetic model. Throughout the treatment, VCS-LT maintained slightly lower FBG levels than acarbose, which indicated a sustained glucose-lowering effect. No apparent hypoglycemic tolerance developed in the VCS-LT group, thus supporting its long-term safety for regular dietary intake.

#### 4.2.3. Postprandial Glycemic Control by VCS-LT Confirmed by OMTT

In this study, the molecular docking results showed favourable interactions with both the MGAM-C and MGAM-N domains. Consistent with these results, the OMTT demonstrated a 16.93% reduction in postprandial glucose exposure after maltose loading. In addition, the FBG results showed that VCS-LT may regulate both fasting and postprandial glycemia in diabetic mice. This effect may occur through intestinal α-glucosidase inhibition, followed by delayed carbohydrate hydrolysis and glucose absorption.

The in vitro pNPG-based assay, in which a yeast-derived enzyme was used, demonstrated that VCS-LT possesses general α-glucosidase inhibitory activity. The molecular docking results further predicted its binding to human MGAM catalytic domains. The OMTT using maltose, which is a substrate specifically hydrolyzed by intestinal MGAM, provided functional in vivo evidence supporting the involvement of intestinal glucosidase inhibition. Taken together, these complementary approaches suggest that VCS-LT likely exerts its hypoglycemic effect through the inhibition of intestinal glucosidases, including MGAM. However, direct confirmation using purified human MGAM or intestinal brush-border membrane preparations is warranted in future studies.

#### 4.2.4. Limitations of the Study

This study had several limitations that should be acknowledged. First, only a single dose of VCS-LT (100 mg/kg) was evaluated in vivo. Dose–response studies are therefore required to determine its efficacy across a broader range and to establish the optimal concentration. Second, the mechanism of action was primarily investigated using molecular docking and enzyme inhibition assays. While these approaches provide supportive evidence, direct binding between VCS-LT and MGAM needs to be confirmed through biophysical methods, such as surface plasmon resonance or isothermal titration calorimetry. Third, we did not perform direct enzymatic assays using purified human MGAM or intestinal brush-border membrane vesicles. The specificity of VCS-LT for MGAM versus other intestinal glucosidases, e.g., sucrase-isomaltase, thus remains to be determined. Fourth, the pharmacokinetic profile of VCS-LT has not yet been characterised, and its long-term safety therefore needs to be established. Its absorption, distribution, metabolism, and excretion, along with extended safety evaluations, should be examined in future works. Fifth, no sensory evaluation, flavour profiling, or consumer acceptability studies have been conducted for VCS-LT in food matrices. These will be essential prerequisites for its eventual application in commercial herbal teas, dietary supplements, and/or functional food products.

Despite these limitations, the present findings offer preliminary evidence supporting the potential of VCS-LT as a food-derived AGI. Most previous studies on *Uncaria* have concentrated on medicinal extracts, and less attention has been paid to its dietary applications. Our study thus contributes to this underexplored area through the evaluation of the purified alkaloid VCS-LT for its α-glucosidase inhibitory activity and its relevance to dietary glycemic control. Nonetheless, further investigations are needed to confirm the efficacy and safety of VCS-LT in actual food systems before it can be considered for incorporation into functional foods or daily dietary supplements.

### 4.3. Prospects for VCS-LT as a Functional Food Ingredient

The food-derived origin and favourable safety profile of VCS-LT support its potential as a functional ingredient for glycemic management. Its demonstrated α-glucosidase inhibitory activity and low effective dose make it suitable for incorporation into herbal teas, low-glycemic grain products, and dietary supplements for populations with prediabetes. Unlike synthetic agents, VCS-LT is derived from *U. rhynchophylla*, a plant with documented use in foods, which may favour its acceptance in routine dietary applications.

To facilitate its translation into commercial products, future food-oriented studies should examine the thermal and pH stability of VCS-LT during processing, its compatibility with various food matrices, and the optimisation of extraction procedures for scalable production. Moreover, systematic sensory evaluation, flavour masking (if necessary), and consumer preference testing in relevant food vehicles, such as herbal teas, cereal bars, or beverages, are critical next steps to ensure that the addition of VCS-LT does not adversely affect the organoleptic properties and consumer acceptance of the relevant foods. Future pharmacokinetic investigations are also warranted. While the primary hypoglycemic action of VCS-LT occurs locally in the intestinal lumen through α-glucosidase inhibition—analogous to acarbose—systemic bioavailability data would be valuable for understanding its overall disposition and safety profile. Notably, related indole alkaloids from *U. rhynchophylla* have demonstrated oral absorption in rodent models [27], which supports the feasibility of future PK studies for VCS-LT.

The recommended investigations will help determine whether the observed bioactivity of VCS-LT can be retained in finished formulations and may provide a basis for developing VCS-LT-containing functional foods.

## 5. Conclusions

In the present study, VCS-LT, an indole alkaloid from *U. rhynchophylla*, inhibited α-glucosidase in vitro and reduced postprandial glucose excursions in vivo. The in vitro data, molecular docking, and OMTT collectively indicated that VCS-LT targets intestinal MGAM via its two catalytic domains. Notwithstanding, direct enzymatic confirmation using human MGAM is still needed. At the tested doses, VCS-LT maintained glycemic control with no adverse effects or hypoglycemia. These findings expand those of previous *Uncaria* research, which has primarily focused on medicinal extracts, by demonstrating that a purified alkaloid can function as a dietary active ingredient. VCS-LT may thus have applications in hypoglycemic teas, low-GI grain products, and nutraceuticals. However, prior to the commercial application of VCS-LT, its use in food will require additional studies that address its processing stability, matrix compatibility, extraction scale-up, and selectivity against other carbohydrate-digesting enzymes, as well as systematic toxicological evaluations and regulatory safety clearance, e.g., GRAS status. Overall, the current findings support further food-oriented research on VCS-LT as a plant-based ingredient for glycemic management.

## Figures and Tables

**Table 1:** Inhibition rate of α-glucosidase by acarbose.

Concentration (μg/mL)	1	10	50	100	500	1000	2000
Inhibition rate (%)	24.03	25.65	30.00	39.68	52.74	55.27	66.34

**Table 2:** Inhibition rate of α-glucosidase by VCS-LT.

Concentration (μg/mL)	1	10	50	100
Inhibition rate (%)	35.55	36.85	36.43	47.47

**Table 3:** Binding energies of VCS-LT and acarbose with MGAM.

Compound	MGAM-C Binding Energy (kcal/mol)	MGAM-N Binding Energy (kcal/mol)
Acarbose	−7.1	−7.9
VCS-LT	−9.5	−9.1

**Table 4:** Body weight changes of the mice presented as mean ± standard deviation (SD).

Group	Body Weight (g)				
	Baseline	Day 0	Day 2	Day 6	Day 11
Control	28.98 ± 1.42 **^++^	32.37 ± 1.64 **^++^	29.33 ± 1.75 *^++^	39.83 ± 2.48 **^++^	43.33 ± 3.14 **^++^
Model	24.17 ± 2.52 ^##^	24.33 ± 3.86 ^##^	25.00 ± 3.95 ^##^	26.67 ± 6.53 ^##^	28.00 ± 7.80^##^
Acarbose	24.22 ± 1.70 ^##^	23.97 ± 1.65 ^##^	26.33 ± 2.80 *^##^	27.97 ± 3.03 *^##^	29.00 ± 3.85 *^##^
VCS-LT	27.22 ± 1.59 *	27.13 ± 2.96 **^##^	31.17 ± 3.06 **^++^	32.77 ± 5.31 **^##++^	34.00 ± 5.37 **^##++^

Mean ± SD (*n* = 6). * *p* < 0.05, ** *p* < 0.01 vs. model; ^##^ *p* < 0.01 vs. control; ^++^ *p* < 0.01 vs. acarbose.

## Data Availability

The original contributions presented in this study are included in the article/Supplementary Materials. Further inquiries can be directed to the corresponding authors.

## Supplementary Materials

The following supporting information can be downloaded at: https://www.mdpi.com/article/10.3390/foods15183329/s1, Figure S1: schematic experimental timeline.

## Abbreviations

The following abbreviations are used in this manuscript:

AGIs	α-glucosidase inhibitors
ALX	alloxan
AUC	area under the curve
DM	diabetes mellitus
DMSO	dimethyl sulfoxide
FBG	fasting blood glucose
IC_50_	half-maximal inhibitory concentration
i.p.	intraperitoneal
MGAM	maltase-glucoamylase
MGAM-C	maltase-glucoamylase C-terminal domain
MGAM-N	maltase-glucoamylase N-terminal domain
OMTT	oral maltose tolerance test
OD	optical density
PBS	phosphate-buffered saline
PDB	Protein Data Bank
pNPG	4-nitrophenyl-α-D-glucopyranoside
SD	standard deviation
T2DM	type 2 diabetes mellitus
VCS-LT	vincoside lactam

## References

[1] International Diabetes Federation (2025). IDF Diabetes Atlas.

[2] Mushtaq A., Azam U., Mehreen S., Naseer M.M. (2023). Synthetic α-glucosidase inhibitors as promising anti-diabetic agents: Recent developments and future challenges. Eur. J. Med. Chem..

[3] Ali T., Mishra G.P. (2026). Recent Advances in Phytoconstituent-Based Alpha-Glucosidase Inhibitors and Therapeutic Potential for Type 2 Diabetes Mellitus. Indian J. Pharm. Educ. Res..

[4] Salehi B., Ata A., V. Anil Kumar N., Sharopov F., Ramírez-Alarcón K., Ruiz-Ortega A., Abdulmajid Ayatollahi S., Valere Tsouh Fokou P., Kobarfard F., Amiruddin Zakaria Z. (2019). Antidiabetic Potential of Medicinal Plants and Their Active Components. Biomolecules.

[5] Dirir A.M., Daou M., Yousef A.F., Yousef L.F. (2022). A review of alpha-glucosidase inhibitors from plants as potential candidates for the treatment of type-2 diabetes. Phytochem. Rev..

[6] Raza A., Abdullah F.N.U., Wahid A. (2025). Assessment of digital technologies in agriculture with particular reference to Digital Dera Initiative in Punjab, Pakistan. Agrobiol. Rec..

[7] Zhao X.M. (1998). Bencao Gangmu Shiyi (Supplement to the Compendium of Materia Medica).

[8] Sukhonthara S., Thipnate P. (2026). Evaluation on bioactive compounds, antioxidant, anti-α-glucosidase and anti-acetylcholinesterase in different parts of *B. flabellifer* L. Int. J. Agric. Biosci..

[9] Idohou R., Satowakou E., Osseni A.A., Gbenou P. (2025). Ethnobotanical knowledge and prioritization of indigenous food plants around the Wari-Maro classified forest in Benin (West Africa). Agrobiol. Rec..

[10] Yuan D., Ma B., Wu C., Yang J., Zhang L., Liu S., Wu L., Kano Y. (2008). Alkaloids from the leaves of *Uncaria rhynchophylla* and their inhibitory activity on NO production in lipopolysaccharide-activated microglia. J. Nat. Prod..

[11] Du T., Yang L., Xu X., Shi X.F., Xu X., Lu J., Lv J.L., Huang X., Chen J., Wang H.Y. (2019). Vincamine as a GPR40 agonist improves glucose homeostasis in type 2 diabetic mice. J. Endocrinol..

[12] Ahmad R., Hashim H.M., Noor Z.M., Ismail N.H., Salim F., Lajis N.H., Shaari K. (2011). Antioxidant and Antidiabetic Potential of Malaysian *Uncaria*. Res. J. Med. Plant.

[13] Triadisti N., Elya B., Hanafi M., Hashim N.M., Illahi A.D. (2025). α-Glucosidase inhibitor compounds of *Uncaria sclerophylla* leaves’ most active chromatography fraction: In vitro, in silico, and ADMET analysis. J. Appl. Pharm. Sci..

[14] Pan G., Lu Y., Wei Z., Li Y., Li L., Pan X. (2024). A review on the in vitro and in vivo screening of α-glucosidase inhibitors. Heliyon.

[15] Tolmacheva T.A., Gasparyan S.V., Nugmanov A.K.-K. (2024). Marketing Research of the Confectionery Sugar Products Market and Improvement of the Technology of Prebiotic Fillings from Non-Traditional Raw Materials. Int. J. Agric. Biosci..

[16] Dewalkar L.P. (2026). Experimental models in diabetes research. Lab. Anim. Res..

[17] Gaulton G.N., Schwartz J.L., Eardley D.D. (1985). Assessment of the diabetogenic drugs alloxan and streptozotocin as models for the study of immune defects in diabetic mice. Diabetologia.

[18] Nguyen T.T., Ryu H.J., Lee S.H., Hwang S., Cha J., Breton V., Kim D. (2011). Discovery of novel inhibitors for human intestinal maltase: Virtual screening in a WISDOM environment and in vitro evaluation. Biotechnol. Lett..

[19] Sim L., Willemsma C., Mohan S., Naim H.Y., Pinto B.M., Rose D.R. (2010). Structural basis for substrate selectivity in human maltase-glucoamylase and sucrase-isomaltase N-terminal domains. J. Biol. Chem..

[20] RCSB Protein Data Bank. https://www.rcsb.org/.

[21] Elsner M., Gurgul-Convey E., Lenzen S. (2006). Relative importance of cellular uptake and reactive oxygen species for the toxicity of alloxan and dialuric acid to insulin-producing cells. Free Radic. Biol. Med..

[22] Huang K., Chen X., Li S., Zhang X., Zhang Y., Zhang Y. (2025). Indole Alkaloids from *Uncaria rhynchophylla* and Their Inhibitory Activities against α-Glucosidase. Phytochemistry.

[23] Rosas-Ramírez D., Escandón-Rivera S., Pereda-Miranda R. (2018). Morning glory resin glycosides as α-glucosidase inhibitors: In vitro and in silico analysis. Phytochemistry.

[24] Wang Y.-H., Liu T.-W., Hsiao S.-W., Chu M.-H., Lee T.-H., Hsu S.-J., Chen S.-Y., Lee C.-K. (2025). Comparative Metabolomics of Acetylcholinesterase and α-Glucosidase Inhibitors in Pericarp of *Garcinia mangostana* L. Bot. Stud..

[25] Hafez Ghoran S., Abdjan M.I., Kristanti A.N., Aminah N.S. (2025). Insights into In Vitro and In Silico Studies of α-Glucosidase Inhibitors Isolated from the Leaves of *Grewia optiva* (Malvaceae). Int. J. Biol. Macromol..

[26] Xu X., Shan B., Liao C.-H., Xie J.-H., Wen P.-W., Shi J.-Y. (2015). Anti-Diabetic Properties of *Momordica charantia* L. Polysaccharide in Alloxan-Induced Diabetic Mice. Int. J. Biol. Macromol..

[27] Jiang S., Yang X., Wang Z., Gan C., Huang J., Sun J., Peng H., Wei F., Wang Z., Yang C. (2022). Biotransformation and Pharmacokinetic Studies of Four Alkaloids from *Uncaria rhynchophylla* in Rat Plasma by Ultra-Performance Liquid Chromatography with Tandem Mass Spectrometry. J. Pharm. Biomed. Anal..

