# Supplementary material for: Vincoside Lactam from Edible Uncaria rhynchophylla: A Food-Derived Indole Alkaloid for Dietary Postprandial Glycemic Regulation and Functional Food Development
Source: Foods. 2026 Sep 20;15(18):3329. doi: 10.3390/foods15183329 (PMC13606520; doi:10.3390/foods15183329)
Supplement: Supplementary file 1 [file foods-15-03329-s001.zip › foods-4537611-supplementary.pdf]

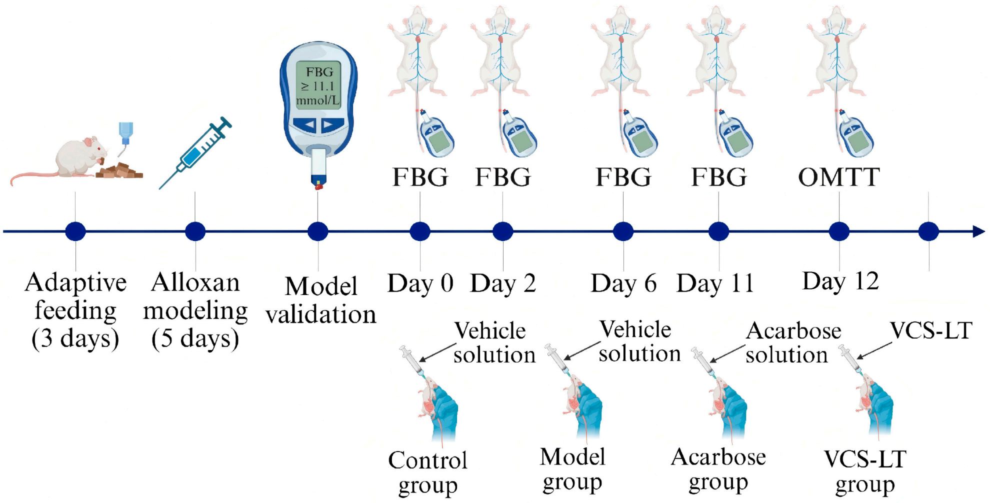

Figure S1: schematic experimental timeline.
